# Expression of Ksp-cadherin during kidney development and in renal cell carcinoma

**DOI:** 10.1038/sj.bjc.6602597

**Published:** 2005-05-10

**Authors:** C Thedieck, M Kuczyk, K Klingel, I Steiert, C A Müller, G Klein

**Affiliations:** 1 Section for Transplantation Immunology and Immunohematology (ZMF), University Medical Clinic, Tübingen, Germany; 2 Department of Urology, University Medical Clinic, Tübingen, Germany; 3 Department of Molecular Pathology, University Medical Clinic, Tübingen, Germany

**Keywords:** cadherin superfamily, cadherin-16, cell adhesion, renal cell carcinoma, renal development

## Abstract

Cadherins are a large family of cell–cell adhesion molecules acting in a homotypic, homophilic manner that play an important role in the maintenance of tissue integrity. In the human kidney, several members of the cadherin family (including E- and N-cadherin, cadherin-6, -8 and -11) are expressed in a controlled spatiotemporal pattern. Cadherin-16, also called kidney-specific (Ksp-) cadherin, is exclusively expressed in epithelial cells of the adult kidney. In renal cell carcinomas (RCCs), which are considered to originate from epithelial kidney tubular cells, a complex pattern of cadherin expression can be observed, but Ksp-cadherin expression has not been analysed so far. In the present study, we show that the expression of Ksp-cadherin is completely abrogated in RCCs. Whereas Ksp-cadherin can be detected at later stages of tubulogenesis during human renal development and in the distal tubules of adult kidneys, no expression was found by immunohistochemistry or Western blot analysis in RCC tumour tissues and several RCC cell lines. However, despite the lack of protein expression, mRNA synthesis of Ksp-cadherin could be detected by reverse transcriptase–polymerase chain reaction analysis in all RCC tissues and most of the RCC cell lines studied, although at a reduced level. The loss of Ksp-cadherin protein was only observed in the malignant part of the tumour kidneys, whereas in the normal part of the affected kidneys Ksp-cadherin expression was clearly detected. These results indicate a downregulation of Ksp-cadherin in RCC and suggest a role for this cell adhesion molecule in tumour suppression.

The incidence of renal cell carcinoma (RCC) has increased steadily during recent years and accounts today for 3% of all solid malignancies (Mulders and De Mulder, 2002). Renal cell carcinomas are often associated with a poor prognosis due to early metastasis which remains the predominant clinical problem for therapeutical intervention in this type of cancer. Although several new therapeutical modalities, for example, immunotherapy and cytotherapy, have been developed over the years for metastatic RCC patients, the prognosis, however, is still very pessimistic for these patients (Oya and Murai, 2003). Dissemination of tumour cells, which finally leads to clinically detectable metastases, is certainly assisted by downregulation of cell adhesion molecules, for example, members of the cadherin family.

Classical cadherins are a gene family of membrane-anchored cell adhesion molecules which can be classified into two subfamilies, namely type I (E-, N-, P- and R-cadherin) and type II (cadherin-5 to -12, -14 and -15) cadherins (Kemler, 1992; Nollet *et al*, 2000). They are not only involved in Ca^2+^-mediated homophilic adhesive cell–cell interactions, but also in transducing external signals into the cells (Wheelock and Johnson, 2003). They are well known to play important roles during embryonic development and in cell differentiation, and also in the maintenance of adult tissue integrity. Both type I and type II cadherins share a characteristic structure consisting of five or more ectodomains, a transmembrane segment and a conserved cytoplasmic tail which interacts via different catenins with the cytoskeleton (Wheelock *et al*, 1996). However, there are a few classical cadherins (which are not classified as type I or type II) with a highly truncated cytoplasmic domain which cannot interact with catenins. One of these cadherin molecules is cadherin-16 also known as Ksp-cadherin (*k*idney *sp*ecific) (Thomson *et al*, 1995; Igarashi, 2003). It is the only member of the cadherin family that is exclusively found in the kidney. A kidney epithelial cell-specific promoter is responsible for the restricted expression pattern of Ksp-cadherin in renal tubular epithelial cells (Whyte *et al*, 1999). In rabbit kidneys, Ksp-cadherin was found on the basolateral membranes of both proximal and distal tubules, but the most prominent expression pattern was seen on distal tubules and collecting ducts (Thomson and Aronson, 1999).

E-cadherin expression is restricted to the distal tubules and collecting ducts of the human kidney, whereas N-cadherin and cadherin-6 expression are found on proximal tubules (Nouwen *et al*, 1993; Paul *et al*, 1997). Cadherin-8 can only be detected on developing tubular structures (Blaschke *et al*, 2002). This rather complex pattern of cadherin expression in the normal adult kidney is mirrored by cadherin expression in RCC (Tani *et al*, 1995; Heicappell, 1999). More than 70% of all RCCs are clear cell carcinomas that are thought to originate from proximal tubular epithelial cells. Therefore, most of the analysed RCC cell lines synthesised N-cadherin and cadherin-6 (Shimazui *et al*, 1996; Blaschke *et al*, 2002). Expression of cadherin-6 can serve as a prognostic marker for tumour progression (Shimazui *et al*, 2003, 2004; Paul *et al*, 2004). Some RCC cell lines also express E-cadherin and cadherin-8 (Shimazui *et al*, 1996; Blaschke *et al*, 2002), but since these cadherins are only found to be expressed on distal tubules or during development, respectively, their expression in the renal tumour cells must be regarded as an aberrant expression pattern.

Although Ksp-cadherin is the only tissue-specific cadherin of the kidney, its expression pattern in RCC has not been studied in detail so far. To analyse Ksp-cadherin expression in the human kidney, reverse transcriptase–polymerase chain reaction (RT–PCR) analyses of embryonic kidney RNA and immunohistochemistry of embryonic and adult kidneys were performed. The mRNA expression pattern in normal renal tissues and in tumour tissues was compared by *in situ* hybridisation and by quantitative and qualitative RT–PCR analyses. By immunohistochemistry and Western blot analysis, the Ksp-cadherin protein expression of 11 RCC cell lines and of native tumour tissue was analysed and compared to the expression within the normal part of the affected kidneys, since alterations in the expression pattern of Ksp-cadherin may be helpful for early diagnosis of RCC.

## MATERIAL AND METHODS

### Renal carcinoma cell lines and tissues

The following human RCC cell lines were obtained from ATCC (Manassas, VA, USA): A-498 (HTB-44), Caki-2 (HTB-47), ACHN (CRL-1611), 786-O (CRL-1932) and 796-P (CRL-1933). The RCC cell lines MZ-1257, MZ-1774 and MZ-1851 were kindly provided by Dr B Seliger (University of Mainz, Germany). Three other RCC cell lines (TW-33, BN-30 and NH-99) were established in the laboratory of Professor Gerhard Müller (University of Göttingen, Germany). All cell lines were grown in culture as described recently (Blaschke *et al*, 2002).

Renal tissue samples were obtained at the time of surgery from 13 RCC patients and from three normal kidneys from the Urology Departments of the Universities of Göttingen and Tübingen and from the Protestant Hospital of Göttingen. Tissue samples were snap frozen in liquid nitrogen and subsequently used for RT–PCR, Western blot analysis or immunohistochemistry. The histology of the tumour specimens is indicated in Table 1.

### Reverse transcriptase–polymerase chain reaction

Total RNA was extracted from the RCC cell lines and from different kidney tissues using the Trizol reagent (Gibco BRL, Eggenstein, Germany). Isolated RNA was purified with RNeasy columns (Qiagen, Hilden, Germany). RNA was further purified by *DNase*I digestion (Pharmacia, Freiburg, Germany). Total RNAs of foetal kidneys from gestation weeks 6, 9 and 12 were commercially available from ViroGen (Watertown, MA, USA).

Kidney-specific cadherin mRNA expression was analysed using a two-step RT–PCR procedure. For cDNA synthesis, 1 *μ*g RNA was transcribed with SuperScript™ First-Strand synthesis system (Invitrogen, Karlsruhe, Germany). In total, 2 *μ*l of the obtained cDNA were subjected to RT–PCR analysis using AmpliTAQ polymerase (Roche Applied Biosystems, Mannheim, Germany). cDNA quality was checked by PCR for detection of the house-keeping gene *β*-actin. Primer sequences for *β*-actin were as follows: *β*-actin (forward primer) 5′-TCA GAA GGA TTC CTA TGT GGG C; reverse primer 5′-CCA TCA CGA TGC CAG TGG TA (product size 317 bp). Primer sequences for Ksp-cadherin, designed according to the sequence NM_004062 (GenBank) were as follows: forward 5′-CAA GTC ATG AGG TGG TGG TG, reverse 5′-TCA TCT GTA TCC TGG GCC TC (product size 358 bp). cDNA samples were amplified by 35 cycles at 94°C for 40 s (denaturation), 60°C for 40 s (annealing) and 72°C for 1 min (elongation), followed by a final elongation step at 72°C for 10 min. Each PCR analysis included distilled water instead of cDNA as negative control. Polymerase chain reaction products were sequenced by cycle sequencing in both directions using the BigDye® Terminator v3.1 Cycle Sequencing kit (Roche Applied Biosystems) to control the specificity of the assay.

### Real-time PCR

For real-time PCR experiments, 1 *μ*g of RNA was reverse-transcribed with iScript™ cDNA Synthesis Kit (BioRad, Munich, Germany) according to the manufacturer's instructions. Real-Time PCR was performed with iQ™ SYBR® Green Supermix (BioRad) using the iCycler iQ™ Real-Time PCR Detection System (BioRad). The *β*-actin and cadherin-16 primers described above were used at a concentration of 20 nM in a 25 *μ*l reaction volume. Reactions were initially heated for 3 min to 95°C, then 40 cycles of 30 s at 95°C, 30 s at 60°C and 20 s at 72°C were performed. After a final denaturation step of 1 min at 95°C, a melting curve was recorded. Polymerase chain reaction products were additionally analysed on a 2% agarose gel.

Cadherin-16 mRNA was quantified in proportion to *β*-actin, using the Gene Expression Macro™, version 1.1 (BioRad). The expression levels were scaled relative to the sample with the lowest expression level, which was designated a value of 1.

### *In situ* hybridisation of kidney specimen

The Ksp-cadherin PCR product was cloned into the dual promotor vector pCR®II TOPO® (Invitrogen) using the Invitrogen TOPO-TA-cloning system. Correct sequence and the orientation of the insert were determined by sequencing using M13 primers. The plasmid was then linearised by complete digestion at the 5′-end of the inserted PCR-product with the *Not*I restriction enzyme (Fermentas, St Leon-Rot, Germany). The obtained linearised DNA was purified by chloroform/phenol extraction and served subsequently as a template for preparation of single-stranded ^35^S-labelled RNA probes, which were synthesised using SP6 RNA polymerase. Enterovirus-specific single-stranded RNA probes were used as negative controls (Klingel *et al*, 1992).

Frozen sections (5 *μ*m) were dried for 2 h at 37°C and fixed for 20 min in 4% PFA at 4°C. After washing with PBS, sections were dehydrated in graded ethanol series (2 × 5 min 70% ethanol, 2 × 5 min 100% ethanol). Until further use, sections could be stored at −80°C in 100% ethanol.

After rehydration in 100, 70 and 40% ethanol and finally in distilled water, *in situ* hybridisation was performed as described previously (Kandolf *et al*, 1987). In brief, slides were pretreated with 0.2 M HCl, heated and digested with proteinase K. Then, the slides were dehydrated in graded ethanol series, dried over night and hybridised at 42°C for 18 h with 500 ng ml^−1^ ^35^S-labelled RNA. After washing, nonhybridised single-stranded RNA was digested with RNase A as previously described (Klingel *et al*, 1992). Hybridised preparations were autoradiographed with Kodak NBT2 film, exposed for several weeks, and stained with haematoxylin/eosin.

### Immunofluorescence staining and immunohistochemistry of RCC cell lines and tissues

Renal cell carcinoma cell lines grown on chamber glass slides and 5 *μ*m cryostat sections of normal kidney tissues were fixed in acetone for 10 min at −20°C. The fixed cells or tissues were incubated with the anti-Ksp-cadherin antibody (clone 4H6/F9, Zymed Laboratories, San Francisco, CA, USA) diluted 1 : 50 for 1 h at RT. After washing, a Cy3™-conjugated goat anti-mouse IgG antibody (1 : 500, Dianova, Hamburg, Germany) was applied for 1 h. For double immunofluorescence staining, the FITC-conjugated lectin peanut agglutinin (PNA) (Linaris, Wertheim, Germany) specific for distal renal tubules was employed. Cell nuclei were identified by counterstaining with 4′,6-diamidino-2-phenylindol-dihydrochloride (DAPI; 1 *μ*g ml^−1^). Negative controls were performed by omitting the primary antibody.

For immunohistochemistry, cryostat sections were stained for Ksp-cadherin employing the Envision method (Dako, Hamburg, Germany). In brief, sections were fixed with acetone and endogenous peroxidase activity was blocked. Kidney specific-cadherin antibody (diluted 1 : 50) was applied for 30 min. After washing, bound antibodies were detected by applying horseradish peroxidase-labelled polymer conjugated to secondary anti-mouse antibody. Peroxidase activity was visualised by addition of 3,3′-diaminobenzidine substrate solution. Slides were counterstained in Mayer's hemalum solution and mounted in Entellan embedding medium (Merck, Darmstadt, Germany).

Paraffin-embedded foetal kidney tissues from 12th week's gestation were obtained from medically indicated abortions. Paraffin sections were deparaffinised in xylene and rehydrated in a graded ethanol series. Subsequently, the slides were heated three times for 3 min at 400 W in a microwave oven in citrate buffer (1 mM sodium citrate, pH 6.0) to reverse crosslinking of the PFA fixation. Then, the procedure was continued as described for the immunohistochemical staining procedure.

### Immunoblot analysis

Total protein extracts from the RCC cell lines and from different kidney tissues were obtained by incubating cells or tissues for 60 min on ice in extraction buffer containing 1% Triton-X-100, 1% NP-40, 1 mM CaCl_2_, 1 mM MgCl_2_, 150 mM NaCl and 50 mM Tris-HCl pH 7.4. Solubilised proteins were separated on 10% polyacrylamide gels and transferred to nitrocellulose filters. Equal protein loading was controlled by Ponceau S (0.1% w v^−1^ in 1% v v^−1^ acetic acid) staining. Nonspecific protein-binding sites were blocked with 5% skimmed milk powder solution. The filters were probed with the Ksp-cadherin antibody (diluted in 5% skimmed milk powder) directed against the C-terminal part of the molecule. After washing, bound cadherin antibodies were detected by alkaline phosphatase-conjugated goat anti-mouse immunoglobulins (Dako), followed by colorimetric reaction with the Fast-BCIP/NBT system (Sigma, Taufkirchen, Germany).

### Proteasome inhibiton assay

Renal cell carcinoma cell lines were seeded in six-well plates and grown to 80% confluency, after which culture medium with 25 or 50 *μ*M *N*-**A**cetyl-**L**eucin-**L**eucin-**N**orleucin (ALLN, Merck Biosciences, Schwalbach, Germany), which inhibits proteasome and lysosome proteases, was added. After 4, 8 or 24 h of incubation at 37°C, 5% CO_2_ cells were washed thoroughly and lysed directly. Cadherin-16 expression was analysed by subsequent Western Blot analysis.

## RESULTS

### Kidney-specific cadherin in human foetal kidney

**Figure 1:**
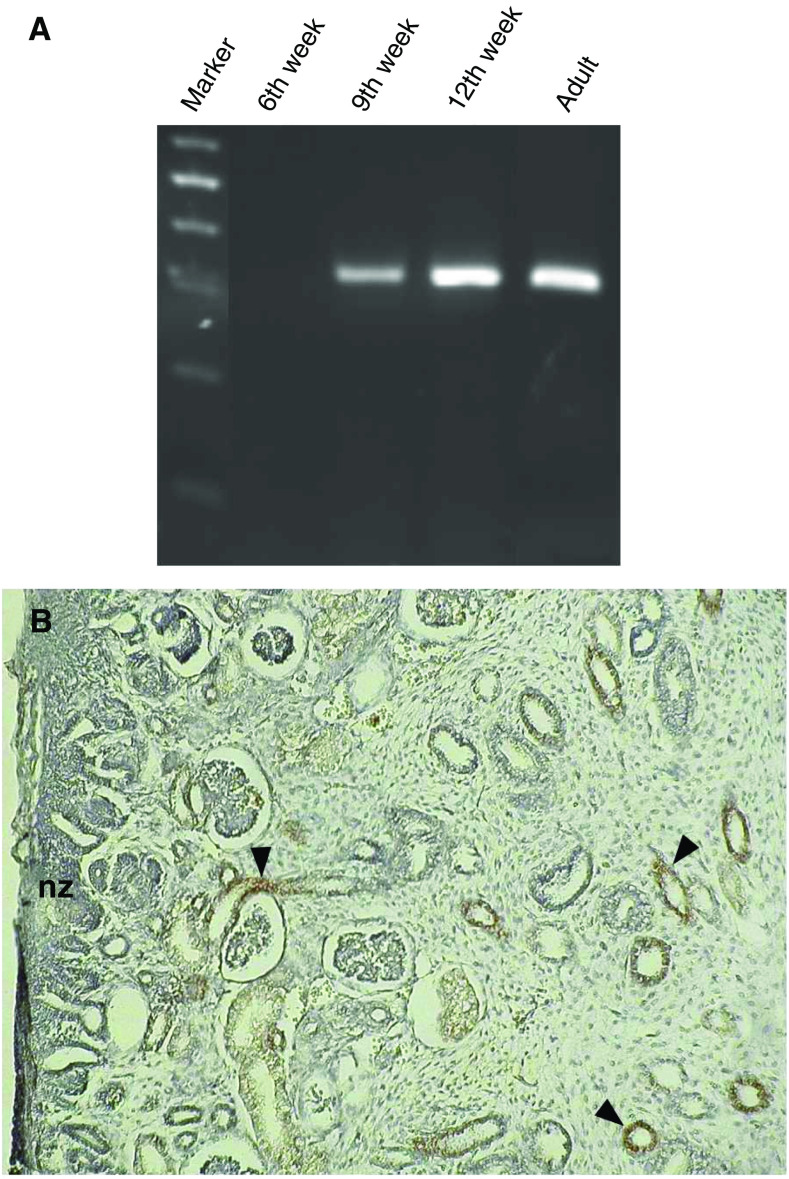
Expression of Ksp-cadherin in foetal kidney. (**A**) Kidney-specific cadherin mRNA expression was studied by RT–PCR analysis with total RNA derived from human embryonic kidneys at 6th, 9th and 12th week's gestation. Kidney-specific cadherin mRNA expression first arises in the 9th week of pregnancy. (**B**) The micrograph shows an immunohistochemical staining of Ksp-cadherin in embryonic human kidney (12th week of gestation). Kidney-specific cadherin was detected in distal tubules and collecting ducts (arrowheads), but not in the early nephrogenic zone (nz).

The expression pattern of Ksp-cadherin during different stages of human kidney development was analysed using RNA of embryonic kidneys of the 6th, 9th and 12th week of gestation. Reverse transcriptase–PCR analyses revealed that Ksp-cadherin expression could not be detected at the earliest stage analysed, but appeared in the 9th week of gestation, increased during further development and was still present in the adult stage (Figure 1A). A control amplification with *β*-actin-specific primers showed specific bands of similar intensity, indicating that equal amounts of intact RNA of all developmental stages were used (data not shown). As revealed by immunohistochemistry, Ksp-cadherin+ cells in a 12th week's embryonic kidney were restricted to the more advanced distal tubules and collecting ducts (Figure 1B). No staining signals were found in the early nephrogenic zone where the transition of the induced mesenchymal cells into the epithelial cells of comma- and S-shaped bodies can be observed.

### Kidney-specific cadherin in adult human renal distal tubules

**Figure 2:**
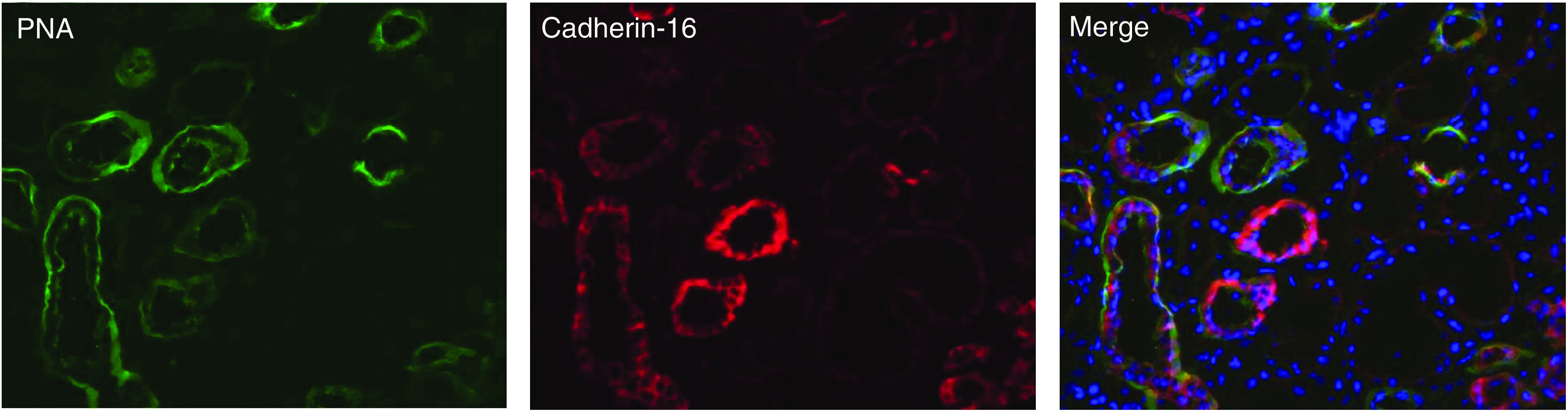
Expression of Ksp-cadherin in adult kidney. The micrographs show immunofluorescence staining with PNA lectin (PNA) and of Ksp-cadherin (cadherin-16) in normal adult kidney tissue of RCC patient 11. The section was counterstained with DAPI to visualise the cell nuclei and the tubules (merge). Ksp-cadherin is present in the tubules stained with PNA lectin, identifying them as distal tubules and collecting ducts, but not in proximal tubules.

In the adult rabbit kidney, Ksp-cadherin expression was detected on all parts of the renal tubules and collecting ducts (Thomson and Aronson, 1999). In the same publication it was reported (but not shown) that this ubiquitous renal tubular expression pattern of Ksp-cadherin is well conserved in human, mouse and rat kidneys. To unambiguously identify the expression pattern in human kidneys, we labelled cryostat sections of normal kidneys with a monoclonal antibody against human Ksp-cadherin and counterstained them with a fluorochrome-conjugated PNA lectin, which specifically recognises epithelial cells of distal tubules and collecting ducts. Double immunofluorescence staining revealed an expression of Ksp-cadherin on distal tubules and collecting ducts, but no expression was found on proximal tubules (Figure 2). Remarkably, tubules with a strong PNA-lectin staining showed relatively weak Ksp-cadherin expression and *vice versa*, suggesting that both markers label different parts of the distal tubules with different intensities.

### Kidney-specific cadherin in RCC

**Figure 3:**
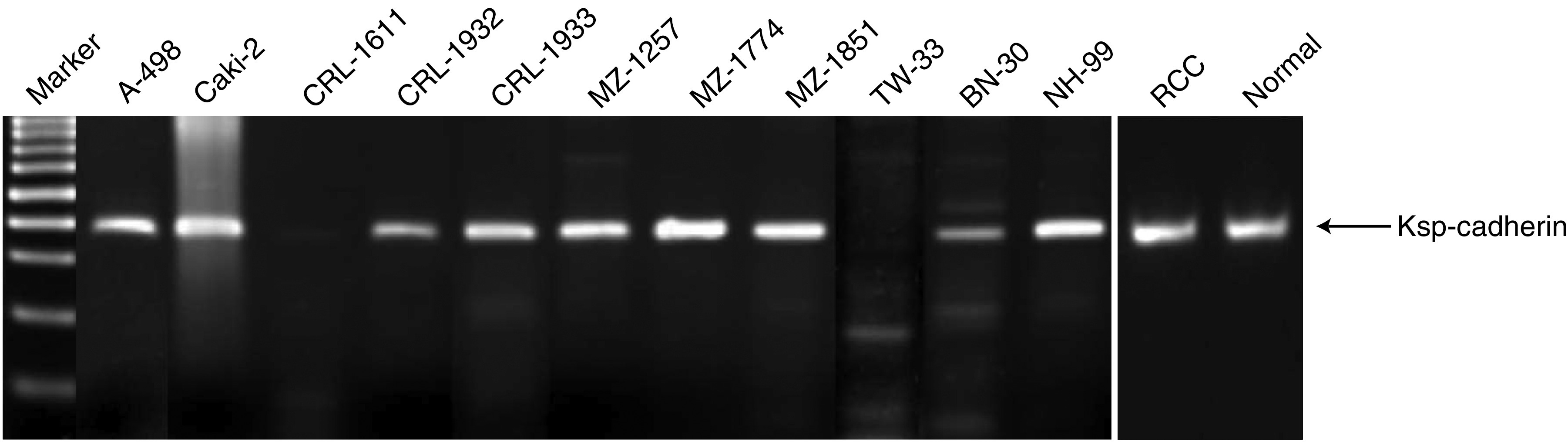
Detection of Ksp-cadherin mRNA expression in RCC cell lines and RCC tissues by RT–PCR analysis. Ksp-cadherin mRNA can be detected in nearly all RCC cell lines except CRL-1611 and TW-33. In RCC and normal kidney tissues, Ksp-cadherin mRNA was also detected.

**Figure 4:**
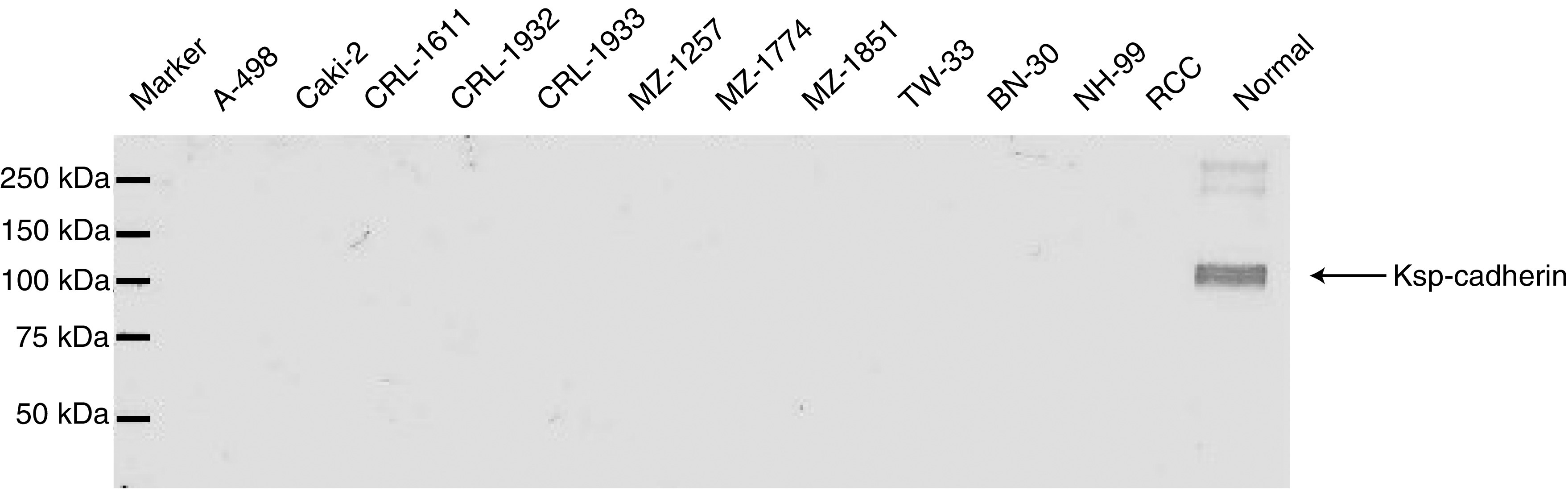
Analysis of Ksp-cadherin protein expression in RCC cell lines and RCC tissues by Western blotting. The Ksp-cadherin protein is only present in the normal kidney, but not in any of the RCC cell lines or RCC tissues studied.

To study Ksp-cadherin expression in RCC, 11 established RCC cell lines were examined by RT–PCR and Western blot analyses. Nine out of 11 RCC cell lines clearly synthesised Ksp-cadherin mRNA (Figure 3). The identity of the 358 bp amplification product, whose sequence is located in exon 13 and exon 14 spanning parts of the extracellular EC5 and EC6 domains of Ksp-cadherin, was verified by cycle sequencing. However, by immunoblotting (Figure 4) and immunofluorescence staining (data not shown), the Ksp-cadherin protein could not be detected in any of the 11 analysed RCC cell lines. To rule out the possibility that the Ksp-cadherin protein is rapidly degraded by the proteasome, RCC cell lines were cultured in the presence of 25 or 50 *μ*M of the proteasome inhibitor ALLN. This treatment did not cause any change in the detection of Ksp-cadherin by Western blot analysis (data not shown) arguing against a proteasomal degradation of the Ksp-cadherin. The expression of Ksp-cadherin mRNA and the absence of the intact protein were also observed in RCC tissue, whereas in normal kidney tissue the mRNA was present and a strong specific signal at 130 kDa could be detected by immunoblotting, indicating that the used anti-human Ksp-cadherin antibody is successfully applicable for this method (Figures 3 and 4).

**Figure 5:**
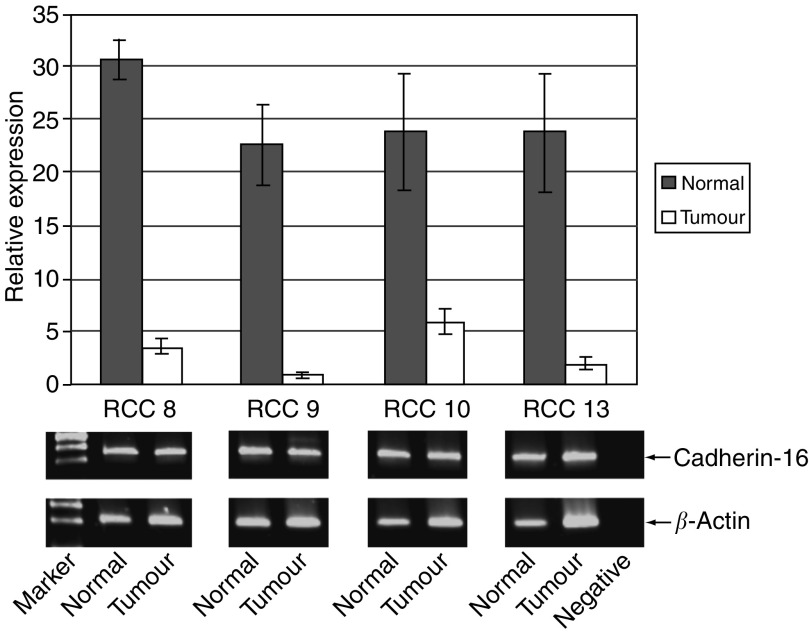
Expression level of Ksp-cadherin mRNA in RCC tissue and the corresponding normal kidney tissues of four RCC patients. Real-time PCR analysis reveals that Ksp-cadherin mRNA expression levels in RCC tissues is 4–23 times lower than in the surrounding normal kidney tissues. However, analysing the end products of real-time PCR after 40 cycles by gel electrophoresis, uniformly strong bands could be observed in normal and tumour tissues of all four (RCC8, RCC9, RCC10, RCC13) analysed patients.

The discrepancy between the lack of Ksp-cadherin protein expression but positive mRNA expression was partially solved by quantitative real-time PCR with RNA from tumour tissues and the corresponding nonaffected parts of the kidneys of four RCC patients. It could be shown that the level of mRNA expression in tumour tissues was 4–23 times lower in comparison to normal tissues (Figure 5). By end point PCR after 40 cycles of amplification, however, clear signals of amplified PCR products were observed both in normal and tumour tissues (Figure 5).

**Figure 6:**
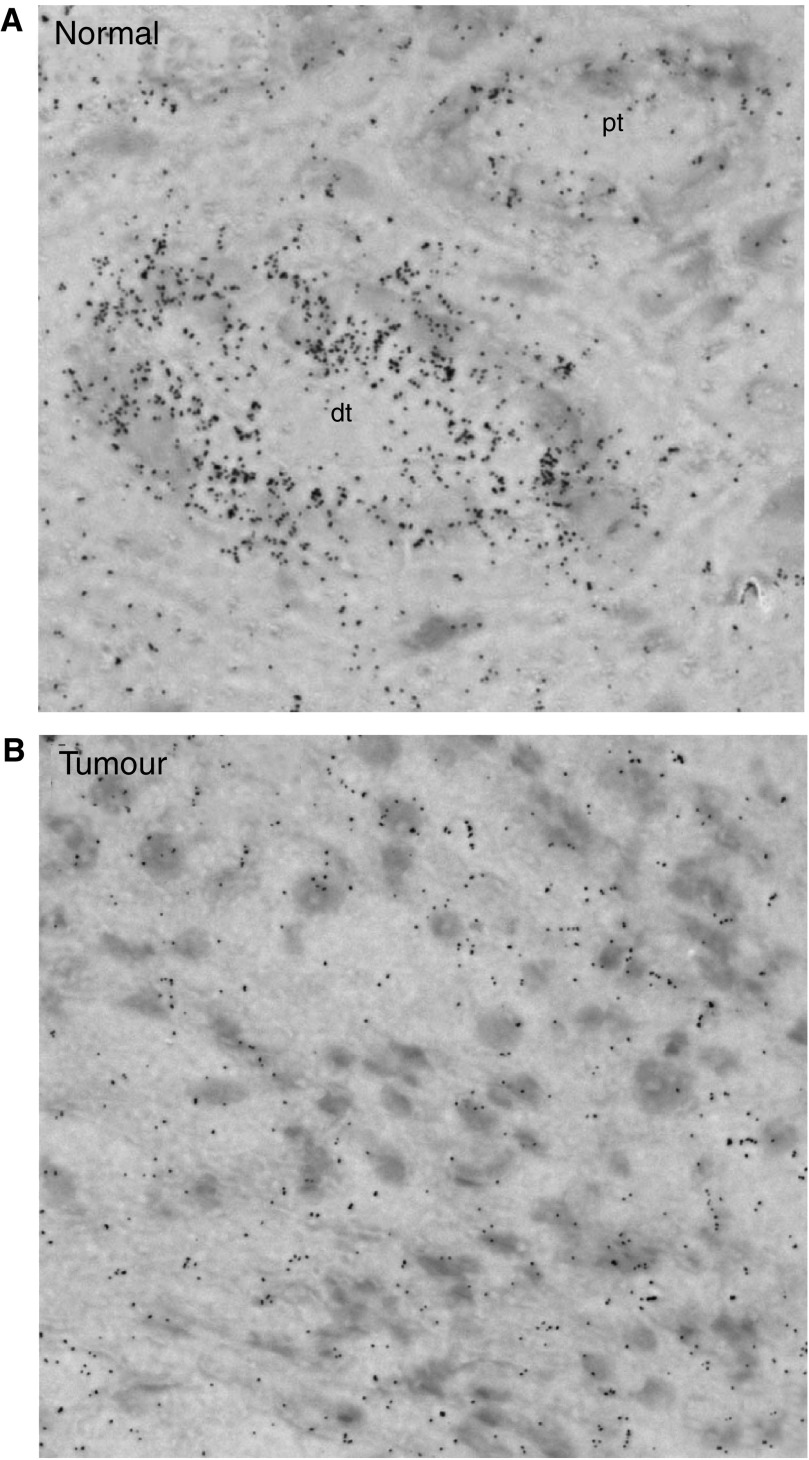
*In situ* detection of Ksp-cadherin mRNA in RCC-tissue and the corresponding normal part of the affected kidney. Frozen sections of the tumour and the normal kidney tissues of 13 patients were hybridised with the ^35^S-labelled cadherin-16-specific antisense RNA probe. Clear signals were only obtained in distal tubular epithelial cells (dt) of the normal kidneys (**A**), whereas signal intensity in proximal tubules (A; pt) and tumour cells (**B**) was not significantly higher than the background.

To analyse mRNA expression of Ksp-cadherin directly in the renal tissue, *in situ* hybridisation experiments with radioactively labelled antisense RNA probes were performed. In the normal kidney, strong signals of Ksp-cadherin mRNA expression could be localised in distal tubules, whereas the signal intensity in proximal tubules was not significantly above background (Figure 6A). In the tumour tissues, which were analysed from 13 RCC patients, the level of signal was not significantly higher than the background labelling (Figure 6B), confirming a low level of Ksp-cadherin mRNA.

**Figure 7:**
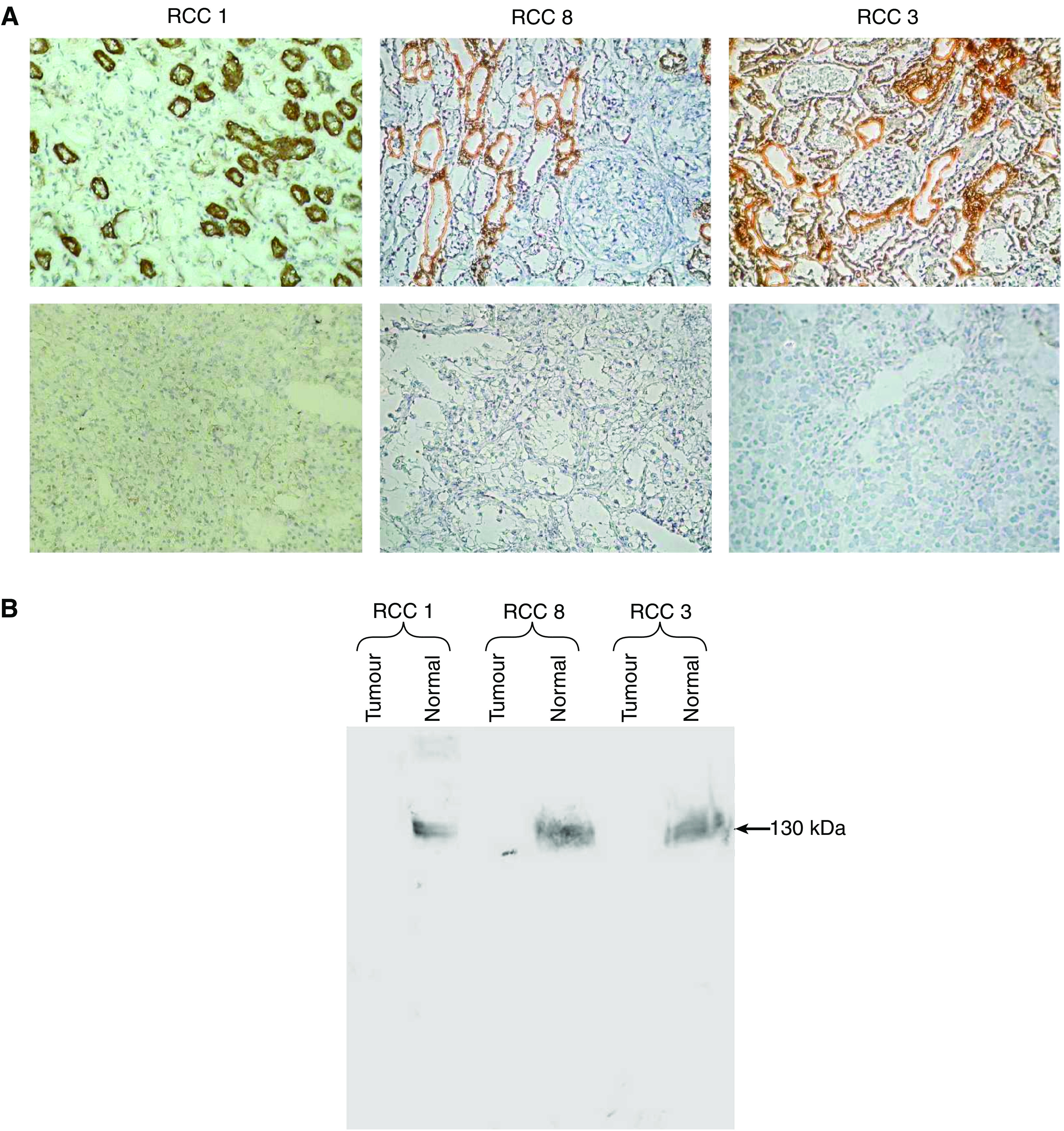
(**A**) Detection of Ksp-cadherin protein expression in RCC tissues and nonmalignantly transformed kidney tissues of the same RCC patients by immunohistochemical staining (**A**) and Western blotting (**B**). Ksp-cadherin can be detected in distal tubules of the normal part of the kidney from patients 1, 3 and 8 (**A**, top panel), but in none of the RCC tissues (**A**, bottom panel). (**B**) The Western blot analysis with lysates from renal tumour tissue and normal parts of the affected kidneys from patients 1, 3 and 8 showed strong specific signals only in the lanes loaded with the lysates from normal kidneys.

The Ksp-cadherin protein expression pattern in 13 tumour tissues and the corresponding normal parts of the kidneys, which were available after total nephrectomy, were also compared by immunohistochemistry and immunoblotting. The RCC1, RCC3 and RCC8 tissues shown in Figure 3 as typical examples were all RCCs of the clear cell carcinoma type. Immunohistochemical staining of the normal parts of all analysed kidneys showed strong staining signals in distal tubules, but no staining was observed in glomeruli and proximal tubules. In the tumour tissues, however, no staining signals at all were detected (Figure 7A). These findings were confirmed with lysates from tumour tissues and the normal, unaffected parts of the kidneys by Western blot analyses. Here, a band of 130 kDa specific for Ksp-cadherin was only found in the lanes loaded with lysates from normal kidneys (Figure 7B).

## DISCUSSION

Ksp-cadherin is an organ-specific member of the cadherin family, which in the human kidney is exclusively found on distal tubules and collecting ducts. During development, Ksp-cadherin does not seem to be involved in the early transition of mesenchymal cells into epithelial cells of the nephron, but appears later in epithelial cells of distal tubules. In RCC tissues and cell lines, the Ksp-cadherin protein cannot be detected, although the mRNA of this cadherin is clearly detectable, albeit at a decreased level, by RT–PCR analysis in tumour cells. Thus, Ksp-cadherin seems to be a further important piece in the puzzling, highly complex expression pattern of the cadherin family in normal and diseased human kidneys.

This first description of Ksp-cadherin expression in human embryonic tissue is in accordance with data obtained in neonatal rabbit kidneys (Thomson and Aronson, 1999), where a relatively late expression of Ksp-cadherin during nephrogenesis has also been observed. The relatively late appearance of Ksp-cadherin during renal development is in contrast to the expression pattern of other cadherins, such as cadherin-8, which can clearly be detected at the 6th week of gestation (Blaschke *et al*, 2002). E-cadherin, which colocalises with Ksp-cadherin in distal tubules of the adult kidney, appears earlier during renal development, directly after the mesenchymal–epithelial transition (Vestweber *et al*, 1985). Thus, it could be speculated that E-cadherin is involved in the initial stages of tubulogenesis, whereas Ksp-cadherin might play a role in stabilisation of terminally differentiated renal distal tubular cells.

In the nephrons of the adult human kidney, Ksp-cadherin expression was observed to be restricted to distal tubules. This was shown by colocalisation with a lectin specifically binding to distal tubules. However, there is an obvious discrepancy between our data and the results obtained by Thomson and Aronson (1999), who reported that Ksp-cadherin can be found on proximal as well as on distal tubules. This discrepancy might be explained by the use of two different antibodies, anti-human Ksp-cadherin antibodies (our present study) and anti-rabbit Ksp-cadherin antibodies (Thomson and Aronson, 1999). The restricted expression pattern of Ksp-cadherin on human renal distal tubules, however, seems to be important in view of the fact that most RCC develop from proximal tubules.

In RCC tissues and cell lines, Ksp-cadherin mRNA could be easily detected by classical end point RT–PCR analysis. Real-time PCR, however, revealed that Ksp-cadherin mRNA can be detected in RCC tumours, but the level of mRNA in the tumour tissues is drastically reduced in comparison to normal tissues. This finding was confirmed by *in situ* hybridisation of Ksp-cadherin mRNA in RCC tissues, where no significant increase above background levels could be was observed. Reverse transcriptase–PCR is a very sensitive and highly specific method, which amplifies even tiny amounts of Ksp-cadherin mRNA in the tumour tissues which cannot be unambiguously detected by *in situ* hybridisation. Thus, the observed, but reduced transcription of the Ksp-cadherin mRNA in RCC tumour tissues and cell lines by RT–PCR without an obvious translation into the Ksp-cadherin protein can be explained in several ways. The most likely explanation is that the amount of Ksp-cadherin mRNA in the tumour tissue is too low to generate enough Ksp-cadherin protein to be detected immunochemically. Secondly, since we have amplified only a part of exon 14 and exon 15 of Ksp-cadherin mRNA, we cannot exclude that a frameshift mutation might have occurred in the RCC cells which cannot be recognised by the RT–PCR analysis. However, since different RCC cell lines and native tumour tissues were analysed, this possibility seems to be unlikely. A third possibility, that the protein is transcribed but rapidly degraded, is also very unlikely since (I) no degradation products were observed by Western blotting and (II) incubation of RCC cell lines with the proteasome inhibitor ALLN did not lead to detectable protein bands by immunoblotting. At the moment we can only conclude that RCC tumour cells express small amounts of Ksp-cadherin mRNA, but no or very low level of detectable protein seems to be translated. However, this knowledge could be used to detect circulating RCC tumour cells by a nested RT–PCR analysis in the periphery of RCC patients, since a kidney cell-specific promoter is responsible for the transcription of Ksp-cadherin mRNA.

Kidney-specific cadherin, as well as E-cadherin, is expressed on distal tubular cells, but most of the RCC cells originate from proximal tubular cells. Therefore, lack of Ksp-cadherin protein expression in RCC tumour tissues seems to be in accordance with the origin of the tumours. However, expression of E-cadherin on RCC tumour cells, which due to the origin must be regarded as an aberrant expression pattern, can be correlated with tumour stages but not with survival rates (Katagiri *et al*, 1995; Shimazui *et al*, 1997). It will be a challenge for the near future to determine in a larger cohort of patients whether the expression of Ksp-cadherin mRNA can also be correlated with different tumour stages or survival rates.

## Figures and Tables

**Table 1:** Analysed RCC tissue specimens

**RCC no.**	**Sex/age**	**Histology**
1	f/73a	Clear cell carcinoma
2	m/58a	Clear cell carcinoma
3	f/64a	Clear cell carcinoma
4	f/85a	Clear cell carcinoma
5	m/56a	Clear cell carcinoma
6	f/71a	Chromophil
7	m/55a	Clear cell carcinoma
8	m/59a	Clear cell carcinoma
9	m/54a	Unknown
10	m/70a	Clear cell carcinoma
11	f/68a	Clear cell carcinoma
12	m/68a	Clear cell carcinoma
13	f/66a	Clear cell carcinoma

RCC=renal cell carcinoma.
